# Could low prolactin levels after radiotherapy predict the onset of hypopituitarism?

**DOI:** 10.1007/s11154-024-09900-1

**Published:** 2024-08-22

**Authors:** Chiara Mele, Stella Pigni, Marina Caputo, Maria Francesca Birtolo, Carola Ciamparini, Gherardo Mazziotti, Andrea Gerardo Antonio Lania, Paolo Marzullo, Flavia Prodam, Gianluca Aimaretti

**Affiliations:** 1 Department of Translational Medicine, University of Piemonte Orientale, Via Solaroli 17, Novara, 28100 Italy; 2 Department of Biomedical Sciences, Humanitas University, Pieve Emanuele (MI), Italy; 3 Endocrinology, Diabetology and Medical Andrology Unit, IRCCS Humanitas Research Hospital, Rozzano, MI Italy; 4 Department of Health Sciences, University of Piemonte Orientale, Novara, Italy

**Keywords:** Prolactin, Hypoprolactinemia, Hypopituitarism, Radiotherapy

## Abstract

Both local and external cranial radiotherapy (RT) can induce neurotoxicity and vascular damage of the hypothalamic-pituitary area, which can promote neuroendocrine alterations. While anterior pituitary insufficiency after RT has been extensively characterized, data on the effect of RT on prolactin (PRL) secretion are limited and heterogeneous, with different patterns of PRL behavior described in the literature. A progressive decline in PRL levels, reflecting a time-dependent, slowly evolving radiation-induced damage to the pituitary lactotroph cells has been reported. To date, the association between hypopituitarism and hypoprolactinemia in patients undergoing RT has not yet been fully investigated. The few available data suggest that lower PRL levels can predict an extent damage of the pituitary tissue and a higher degree of hypothalamic dysfunction. However, most studies on the effect of RT on pituitary function do not properly assess PRL secretion, as PRL deficiency is usually detected as part of hypopituitarism and not systematically investigated as an isolated disorder, which may lead to an underestimation of hypoprolactinemia after RT. In addition, the often-inadequate follow-up over a long period of time may contribute to the non-recognition of PRL deficiency after RT. Considering that hypoprolactinemia is associated with various metabolic complications, there is a need to define appropriate diagnostic and management criteria. Therefore, hypoprolactinemia should enter in the clinical investigation of patients at risk for hypopituitarism, mainly in those patients who underwent RT.

## Introduction

### Hypopituitarism: definition, epidemiology and clinical characteristics

Hypopituitarism is a rare disorder characterized by a complete or partial insufficiency of pituitary hormones [1]. When acquired, this condition can either develop rapidly after surgery, radiotherapy (RT), injuries, vascular accidents, or specific treatments or gradually over months or years [2].

The estimated incidence of congenital hypopituitarism is 1 in 4000-10,000 live births per year [3]. Data on the incidence and prevalence of acquired hypopituitarism are limited and it is probably underdiagnosed when considering the different characteristics at diagnosis, the frequent failure of dynamic tests to diagnose subclinical or transient hypopituitarism, and the lack of international registries [4]. Conventionally, the reported prevalence is 45.5 cases per 100,000 inhabitants and the incidence is 4.2 cases per 100,000 per year, with both figures increasing with increasing patient’s age [5]. Over the last two decades, an increase in the prevalence of hypopituitarism has become evident, possibly due to the widespread use of neuro-radiological tools for non-pituitary indications, with the detection of more pituitary adenomas, and also due to emerging causes of hypopituitarism, such as cancer immunotherapy and traumatic brain injury [2].

Hypopituitarism is, typically, a chronic lifelong disease that is associated with an increased mortality rate, mainly due to cardiovascular, metabolic and respiratory events, when the disease is left untreated. The impact of hypopituitarism depends on the extent and severity of hormonal deficiencies, the age of onset and the duration of the disease. In general, the clinical picture of hypopituitarism is variable as it may involve nonspecific symptoms such as fatigue, anorexia, arthralgia, and headache, or present with life-threatening conditions requiring emergency admission, such as adrenal crisis, water and salt imbalance, or severe hypoglycaemia [6]. Moreover, the clinical presentation varies according to the specific hormonal deficiency and usually reflect the symptoms associated with the loss of peripheral hormones regulated by the hypothalamus-pituitary unit [6–11].

Despite the effort invested to depict the pathophysiology and clinical consequences of hypopituitarism, clinical phenotypes resulting from prolactin (PRL) deficiency still remain under-investigated.

### Causes of hypopituitarism: focus on radiotherapy

With the exception of rare aetiologies of hypopituitarism due to genetic causes [2, 12], the main enlisted causes of acquired hypopituitarism usually depend on direct and indirect mass effects yielded by pituitary tumours or originate from neurosurgical and/or irradiative approach of lesions located within the intrasellar, parasellar and suprasellar area [13, 14]. In addition, hypopituitarism can result from an impaired delivery of hypothalamic hormones to the pituitary gland due to direct mechanical compression of the pituitary stalk or raised intrasellar pressure [15]. Other clinical conditions include acquired brain injury (i.e. traumatic brain injury and stroke), inflammatory and autoimmune diseases, infections and paraneoplastic syndromes [2, 16]. Moreover, innovative cancer treatments with immune checkpoint inhibitors are associated with an increased risk of hypopituitarism [17]. A specific focus on RT-induced hypopituitarism will be reported in the following paragraph.

#### Radiotherapy-induced hypopituitarism

Local and external cranial RT is used for a range of intracranial, base skull and nasopharyngeal tumours as well as haematological neoplasms [18]. RT was first used to treat pituitary adenomas more than 100 years ago and represents a viable alternative for both functioning or non-functioning pituitary adenomas needing growth and/or secretory control for patients who are considered untreatable by surgery, or for those who have been unsuccessfully treated with medical therapy and/or surgery, or as an option for relapsing/expanding tumours [19–22]. Local control of the tumour mass can be achieved in 90–95% of irradiated patients with pituitary tumours within 10–20 years post-RT, while the control of hormone hypersecretion in patients with GH-, ACTH- and PRL-secreting adenomas is variable and reportedly ranges between 40 and 80% at 5 years [23, 24].

Along with their therapeutic effects, both local and external cranial RT can induce undesired neuroendocrine alterations of hypothalamic-pituitary functions [18]. The radiation-related neurotoxicity affecting the hypothalamic-pituitary axes directly depend on the type of RT (conventionally external beam RT or stereotactic RT), the radiation dose, the duration of the radiation treatment and the fraction size [25]. Radiation-induced hypopituitarism is often progressive and irreversible, may affect one or more hypothalamic-pituitary axes and, at higher radiation doses, it often causes the deficit of multiple pituitary hormones [18, 26].

**Fig. 1:**
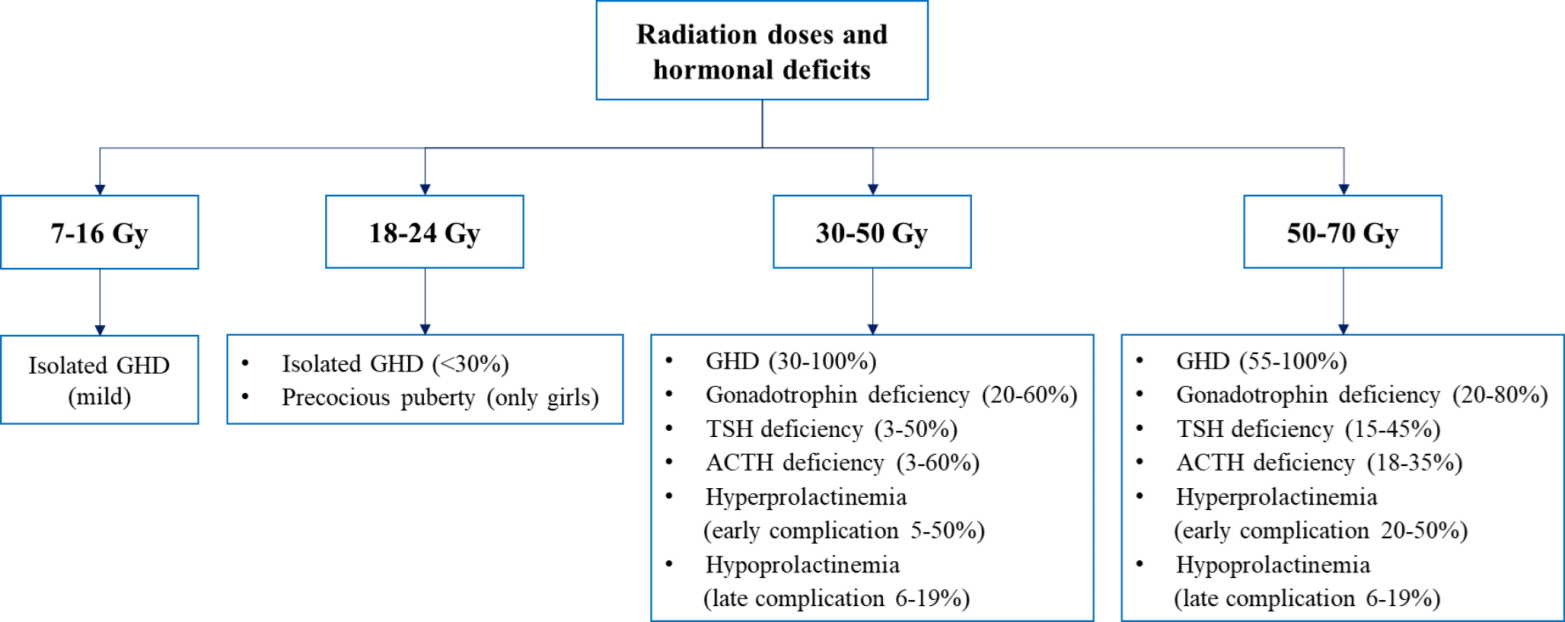
Anterior pituitary hormones abnormalities following RT according to radiation doses

As far as the pathophysiological mechanisms of radiation-induced hypopituitarism are regarded, several evidences point at a direct neuronal damage of the hypothalamus as the main mechanism, while vascular injury could represent a subordinate event [27]. The wide variability in anterior pituitary hormone deficiency rates, with growth hormone (GH) secretion being the most frequently detected, implies a different radiosensitivity of hypothalamic neurons and pituitary cells [25]. Animal models and clinical observations suggest that the somatotropic axis is the most radiosensitive endocrine component, since it can be damaged by lower doses of radiation, like those typically used for subjects with brain tumours or leukemia (10–50 Gy) [28, 29]. Conversely, higher doses (up to 50 Gy) used for persons with pituitary tumours, craniopharyngiomas, skull-base tumours, head and neck tumours, and some non-pituitary brain neoplasia can damage other more radioresistant neuroendocrine cells, indeed leading to multiple hormone deficiencies [30, 31]. Figure 1 summarizes the abnormalities of anterior pituitary hormones following RT according to the radiation doses.

Several factors are related to the development of radiation-induced hypopituitarism, including:

- Younger age: hypopituitarism is more prevalent in children compared to adults, and in younger children as compared to older ones [18];
- Schedule: a shorter period of the radiation administration is associated with a higher risk of hypopituitarism and severity of hormonal deficits [25];
- Time elapsed since treatment: the risk of hypopituitarism increases with time since RT; the progressive decline of hypothalamic-pituitary function following radiation can be explained by pituitary atrophy due to the lack of hypothalamic releasing hormones or delayed direct effects of radiations on the hypothalamic-pituitary cells [32–34]. This latter mechanism is supported by the progressive decrease in the transient hyperprolactinemia observed in subjects after prolonged follow-up periods after RT [35].

While the role of RT in inducing anterior pituitary deficiency has been extensively characterized, the role of low PRL level after RT as a potential predictor of the onset of hypopituitarism has been poorly investigated.

## Hypoprolactinemia: definition, diagnosis and clinical overview

### General aspects

PRL is a polypeptide hormone primarily synthesized and secreted by the pituitary lactotroph cells. Unlike other anterior 55pituitary hormones, the release of which is regulated by specific hypothalamic releasing factors, PRL secretion is predominantly under the inhibitory control of dopamine from the tuberoinfundibular dopaminergic neurons [36]. The main effects of PRL are to stimulate the proliferation and differentiation of the mammary cells (mammogenesis), and to promote the production of milk (lactogenesis) and the maintenance of milk secretion (galactopoiesis) in the postpartum period [36, 37]. Besides its crucial role during pregnancy and lactation, to date more than 300 different pleiotropic effects have been attributed to this hormone, including roles in metabolic and bone homeostasis, reproduction, maternal behaviour, immunomodulation, and regulation of the adrenal function [37–39].

Disorders of PRL secretion can manifest as either hyperprolactinemia or hypoprolactinemia. While hyperprolactinemia, marked by chronically elevated PRL levels, is more widely recognized and has been extensively investigated due to its association with clear clinical manifestations (i.e. galactorrhea and oligo/amenorrhea in women, erectile dysfunction in men, loss of libido and infertility in both sexes), hypoprolactinemia, which is characterized by abnormally low or undetectable PRL levels, remains often overlooked and poorly understood like an endocrine Cinderella [37, 40].

The prevalence of hypoprolactinemia varies depending on the underlying etiology, with congenital forms being far less common than acquired ones. Congenital hypoprolactinemia can result from genetic mutations affecting the normal differentiation and/or function of the pituitary lactotrophs. Most of these genetic forms of PRL deficiency are the consequence of mutations in genes encoding transcription factors crucial for pituitary development which can lead to congenital hypopituitarism, including hypoprolactinemia [15, 41]. Acquired PRL deficiency (APD) can stem from any of the aforementioned causes of hypopituitarism as a consequence of a damage to the lactotrophs. Epidemiological data on the frequency of hypoprolactinemia in this setting are limited, mainly due to underdiagnosis and the lack of standardized diagnostic criteria [40].

### Symptoms and diagnosis of hypoprolactinemia

The most evident clinical manifestation of hypoprolactinemia is the failure of lactation after delivery (puerperal alactogenesis), as typically seen in women with Sheehan Syndrome, PROP-1 mutations or isolated PRL deficiency [37, 40, 42, 43]. Accordingly, the administration of dopamine agonists during pregnancy or in the postpartum period may prevent puerperal lactation [44]. Some authors have also reported a relatively increased risk of failure to initiate breastfeeding and delayed onset of lactogenesis in obese women due to a reduced PRL response to infant suckling [45, 46]. Conversely, no clearly defined symptoms of PRL deficiency have been reported in men as well as in non-lactating and non-pregnant women. Although some anecdotal cases of irregular menstrual cycles, decreased fertility, and early menopause have been described in women with isolated PRL deficiency, a causal relationship cannot be established due to the paucity of data [47]. Such lack of clinically significant consequences of hypoprolactinemia beyond its impact on lactation may explain why PRL deficiency is usually neglected in the diagnosis of hypopituitarism. Indeed, as recently pointed out by Karaca et al., PRL deficiency is not included in the classification of hypopituitarism, and it currently has no impact on its management [40]. Nonetheless, growing evidence suggests that hypoprolactinemia may have some detrimental effects, as it has been associated with increased cardiometabolic risk, sexual dysfunction, and reduced well-being [38, 40, 48–51].

The diagnosis of hypoprolactinemia can be established within the appropriate clinical context and demonstrated by evidence of undetectable or low basal unstimulated serum PRL levels which fail to increase after administration of TRH, insulin, or antidopaminergic agents (e.g. chlorpromazine, metoclopramide), although such dynamic endocrine tests are rarely used for this purpose in routine clinical practice [37, 40, 47]. Currently, there are no universally accepted criteria for the diagnosis of PRL deficiency, as different cut-off both for basal and stimulated PRL levels have been used in different studies (Table 1) [5, 48, 49, 51–61]. In the study by Toledano et al., normal levels of PRL were 5–17 ng/ml for men and 5–20 ng/ml for women according to the reference range of the available immunoassays. The authors classified PRL deficiency as mild when basal PRL levels were 3–5 ng/ml and severe when ˂3 ng/ml [58]. A similar definition of hypoprolactinemia (PRL < 5 ng/ml) was also proposed by Corona and co-authors in a study on male patients with sexual dysfunction [49]. In contrast, Mukherjee et al. defined severe PRL deficiency as a PRL level persistently below the detection limit of the PRL assay (< 2.35 ng/ml) on at least three separate occasions [56]. Diri et al. retrospectively investigated 114 women with Sheehan Syndrome and found that none of the patients with basal PRL levels < 4 ng/ml responded to TRH stimulation test, whereas all patients with basal PRL levels > 7.8 ng/ml showed an adequate response, defined as a doubling of basal PRL levels [59]. On the other hand, some authors proposed gender-dependent thresholds for basal PRL levels that are capable of defining PRL sufficiency in individuals with panhypopituitarism. In fact, these authors found that basal PRL levels ≥ 8.5 ng/ml in males and ≥ 15.2 ng/ml in females could predict adequate PRL responses to TRH stimulation test (defined as a peak PRL response > 18.3 ng/ml in men and > 41.6 ng/ml in women) with 96–100% specificity and 66–76% sensitivity, while PRL ≤ 5.7 ng/ml in males and ≤ 7.1 ng/ml in females predicted inadequate responses with 100% specificity and 70–80% sensitivity [48]. Accordingly, measurement of basal PRL level may be sufficiently informative on hypoprolactinemia in most hypopituitary patients in the routine clinical practice, while the TRH stimulation test, which is no longer available in many countries, could be reserved for equivocal results.

**Table 1:** Criteria used in the diagnosis of hypoprolactinemia in patients investigated for several conditions

Authors	Country	Condition	Gender	PRL cutoff used for deficiency
**Gonzales et al.**,** 1989** [52]	Peru	Fertility	Males	Basal PRL ≤ 6 ng/ml
**Ufearo et al. 1995** [53]	Nigeria	Fertility	Males	Basal PRL < 6.5 ng/ml
**Regal et al.**,** 2001** [5]	Spain	Hypothalamic-pituitary disease	Both	TRH-stimulated PRL response < 3 times the baseline level
**Safer et al. 2013** [54]	US	Psychiatric disorders	Both	Basal PRL ≤ 6 ng/ml
**Sogawa et al.**,** 2016** [55]	Japan	Psychiatric disorders	Both	Basal PRL < 3.57 ng/mL in males and < 6.12 ng/mL in females
**Mukherjee et al. 2003** [56]	UK	Hypothalamic-pituitary disease	Both	Basal PRL < 2.35 ng/ml
**Mukherjee et al. 2006** [57]	UK	Hypothalamic-pituitary disease	Both	Basal PRL < 1.8 ng/ml
**Toledano et al. 2007** [58]	Israel	Hypothalamic-pituitary disease	Both	Basal PRL 3–5 ng/ml (mild) and < 3 ng/ml (severe)
**Corona et al. 2009** [49]	Italy	Sexual dysfunction	Males	Basal PRL < 5 ng/ml
**Diri et al. 2014** [59]	Turkey	Sheehan syndrome	Females	Basal PRL < 4 ng/ml
**Maseroli et al. 2023** [60]	Italy	Sexual dysfunction	Females	Basal PRL < 9.83 ng/ml
**Tasaki et al. 2021** [61]	Japan	Schizophrenia	Males	Basal PRL < 5 ng/ml
**Krysiak et al. 2022** [51]	Poland	Cabergoline induced hypo-prolactinemia	Females	Basal PRL < 5 ng/ml
**Uzun et al. 2024** [48]	Turkey	Hypothalamic-pituitary disease	Both	TRH-stimulated PRL response < 18.6 ng/ml in males and < 41.6 ng/ml in females

Interestingly, following the emerging role of PRL in metabolic homeostasis, a new classification of PRL levels has been proposed, along with the new concept of Homeostatic Functionally Increased Transient Prolactinemia (HomeoFIT-PRL) [62]. Therefore, PRL levels within 7–25 ng/ml should be considered as normal, whereas PRL levels < 7 ng/ml have been suggested as hypoprolactinemia which has been associated with metabolic alterations. On the other hand, while PRL levels > 100 ng/ml should raise suspicion for prolactinoma, moderately high PRL levels (25–100 ng/ml) in absence of recognizable causes of hyperprolactinemia, defined as HomeoFIT-PRL, may represent a physiological response to transient stimuli requiring an increase in metabolic demand (e.g. insulin-induced hypoglycemia, stress, sexual arousal, intense exercise, circadian peaks), aimed to promote metabolic adaptation [62, 63].

### Clinical implications

As mentioned above, PRL has recently been recognized to exert pleiotropic effects, including an intriguing role in metabolism [37, 38, 63]. In rodent pancreatic islets, PRL has been demonstrated to promote β-cell proliferation, insulin production and glucose-induced insulin release [64, 65]. It may also influence energy balance through orexigenic effects on the central nervous system, as well as via effects on adipose tissue differentiation and function, and on lipid metabolism [37, 63, 66]. Noteworthy, clinical studies in humans seem to indicate a U-shaped relationship between PRL levels and cardiometabolic risk, as both hyper- and hypoprolactinemia have been associated with various metabolic complications, including obesity, impaired glyco-insulinemic and lipid profiles, and metabolic syndrome (MetS) [38, 51, 62, 63].

An analysis in males with sexual dysfunction showed that PRL levels in the lowest quartile (˂5 ng/ml) were associated with a higher prevalence of MetS [49, 50]. Particularly, among MetS components, elevated glycemia and elevated triglycerides showed the strongest association with hypoprolactinemia [49]. Similarly, a study of middle-aged and elderly men found low PRL to be associated with an overall unhealthy metabolic phenotype (elevated BMI and glucose levels) and with MetS, as well as with lower levels of physical activity and self-reported general health [50]. A large population-based study in women also documented an inverse association between PRL levels and the risk of developing MetS, although significance was lost after adjustments for covariates and in longitudinal analysis using multivariable regression models [67]. More recently, Krysiak et al. investigated the cardiometabolic profile of young women with iatrogenic hypoprolactinemia resulting from chronic cabergoline treatment [68]. They showed that PRL deficiency, defined here as PRL levels < 5 ng/ml, was associated with increased levels of several cardiometabolic risk factors, including higher levels of 2-h post-OGTT plasma glucose, glycated hemoglobin, triglycerides, uric acid, as well as lower values of HDL-cholesterol and insulin sensitivity. Oppositely, such unfavorable metabolic effects were not observed when PRL levels were maintained within the reference range during treatment and improved after cabergoline dose reduction and normalization of PRL levels [68]. In another study, these authors further investigated the impact of hypoprolactinemia on the effects of atorvastatin in two groups of cabergoline-treated premenopausal women with hypercholesterolemia compared with dopaminergic-naïve normoprolactinemic women matched for age and lipid levels. The authors found that hypoprolactinemia was associated with impaired cardiometabolic effects of atorvastatin, including attenuated lipid-lowering effects on total and LDL cholesterol levels and worsened insulin sensitivity [51].

Similarly, others demonstrated an inverse association between circulating PRL levels and the prevalence of T2DM, as well as a higher risk of developing T2DM in subjects with lower PRL levels, especially in women [68–71]. Of note, lower serum PRL during pregnancy has been associated with a higher risk of postpartum prediabetes/diabetes [72, 73].

Regarding hepatic lipid metabolism, one clinical study found lower PRL levels in patients with Metabolic Dysfunction-Associated Steatotic liver disease (MASLD) compared with non-MASLD controls. Moreover, circulating PRL levels were lower in patients with biopsy-proven severe MASLD as compared with those with mild-to-moderate disease. Results from cell experiments suggested that PRL may improve hepatic lipid accumulation via the CD36 pathway, which is a key transporter of free fatty acid uptake in the liver [74].

Hypoprolactinemia has also been implicated in impaired sexual function in both sexes. In men with sexual dysfunction, low serum PRL levels have been associated with atherogenic erectile dysfunction, premature ejaculation, and anxiety symptoms [49]. Additionally, in European middle-aged and elderly men, low PRL levels were related to several psychological and sexual unhealthy characteristics, including orgasmic difficulties and depressive symptoms [50]. Similarly, PRL-deficient patients with panhypopituitarism showed higher depression scores compared to healthy controls, and lower sexual function scores in males. Remarkably, female sexual functions seemed to be unaffected in this study population [60]. This latter evidence seems to conflict with previous findings in young women indicating that dopamine agonist-induced hypoprolactinemia was associated with impaired sexual functioning, i.e. low sexual desire and arousal, as well as reduced well-being [75]. In a study carried out in pre- and post-menopausal normoprolactinemic women consulting for sexual dysfunction, those with the lowest PRL levels complained of poorer sexual desire than those with the highest PRL levels. These authors suggested that having serum PRL < 9.83 µg/L was predictive of hypoactive sexual desire disorder [60].

In addition to the previous, animal data obtained in vitro and in vivo have shown that PRL is involved in other physiological processes, including immunoregulation, maternal care, neuroprotection, response to stress and adrenal function, and bone metabolism [37, 39, 76]. For instance, data in mice have suggested that an effect of PRL on osteoblasts could be required for normal bone formation and maintenance of bone mass [77]. How these data translate to human remains unanswered. Thus, hypoprolactinemia is potentially linked to a spectrum of clinical effects, yet the underlying mechanisms remain unclear. Whether an increase in the dopaminergic and/or a decrease in the serotoninergic signaling are involved for these effects remain arguable [38, 40, 49, 50]. Further research is needed to clarify the mechanisms underlying these effects and their clinical relevance, especially in patients with hypopituitarism.

## Hypoprolactinema as a form of hypopituitarism

Hypoprolactinemia is associated much less commonly than hyperprolactinemia with hypopituitarism. In exploring the link between low PRL levels and hypopituitarism, a number of conditions relating to different pathophysiological mechanisms have been described:

- ***Genetic conditions affecting pituitary transcription factors***. Appropriate pituitary development is critical for functions relating to metabolic control, bone growth, skeletal muscle development, puberty and reproduction, stress response, lactation and aging. Pituitary organogenesis is strictly regulated during ontogeny and depends on transcription factors (TFs), such as PROP1, POU1F1 (PIT1), HESX1, LHX3 and LHX4 that act at different stages of pituitary development with temporally and spatially organized interactions with DNA and TF co-activators. Mutations altering the expression or function of TFs can instigate variable combinations of pituitary hormone deficiencies, of which those involving PRL deficiency include: recessive homozygous loss-of-function mutations of the LHX3 gene encoding for a LIM-type homeodomain transcription factor [78, 79], homozygous recessive or compound heterozygous recessive mutations in the human Prophet of Pit1 (PROP1) gene [79, 80], recessive and dominant mutations of the functional domains of POU1F1 [81], loss-of function mutations of the Immunoglobulin Superfamily Member 1 (IGSF1) gene causing the X-linked IGSF1 deficiency syndrome, the most common genetic cause of central hypothyroidism [82].
- ***Non-tumoural hypothalamic-pituitary diseases.*** As previously mentioned, a sequential pattern of pituitary hormone loss in hypopituitarism typically comprises GH, gonadotropins, TSH and ACTH, with PRL deficiency developing late and reflecting more severe pituitary dysfunction [10]. In patients with pituitary diseases, hypoprolactinemia shows no race predilection and has similar incidence between males and females. It often remains a disregarded clinical event in terms of clinical outcome (lactation), in terms of diagnostic cut-off (Table 1), and in relation to its diagnostic impact in the setting of GHD [83]. Against the view that hypoprolactinemia is a classical “child of a lesser god” in the setting of hypopituitarism, a recent review paper proposed distinguishing cases of panhypopituitarism into those with normal/high PRL level and those with low PRL level, yet the clinical implication of this sorting remains unestablished [40].
- ***Hypothalamic-pituitary neoplasms.*** In these patients, hypoprolactinemia is rare and more often seen in association with largely invasive tumours and/or robust curative efforts elicited by surgery and RT. As such, in a temporal order of presentation, PRL deficiency is classically the last to develop and severe hypoprolactinemia is rare, but it has been suggested to be a reliable marker of severe hypopituitarism, independently associated with low serum IGF-1 [15]. A retrospective series on 369 patients with hypothalamo-pituitary disease from the Christie’s Hospital [56] documented hypoprolactinemia in 22 cases (6.0%). Most cases had previously undergone neurosurgery (*n* = 19) and/or RT (*n* = 10). No cases of isolated hypoprolactinemia were reported. At variance with the previous, other series reported higher rates of PRL deficiency in patients bearing hypothalamo-pituitary disorders. An analysis conducted in the Spanish region of South Galicia found PRL deficiency in 17% of 69 patients with hypothalamic-pituitary diseases. While this deficiency figure remained lower than that seen for the anterior pituitary axes, 75% of cases diagnosed as hypoprolactinemia had panhypopituitarism [5]. Similar findings were observed in a retrospective analysis on patients subdivided as with normal (> 5 ng/ml), mild (3–5 ng/ml), and severely PRL-deficient (< 3 ng/ml), reporting that 14% and 13% of them were severely and mildly PRL deficient, respectively [72]. An expected parallelism related the severity of hypoprolactinemia to the extent of hypopituitarism, as hormone deficits were more frequently multiple in patients with severe PRL deficiency than in those with normal PRL (71% vs. 37%). The link between hypoprolactinemia and degree of panhypopituitarism has been probably best detailed in a retrospective study of Uzun et al. on a limited sample of 48 patients with anterior panhypopituitarism, 50% of whom had previously undergone surgery [48]. The authors observed a higher prevalence of hypoprolactinemia in subjects with panhypopituitarism, suggesting that APD could represent a marker of more severe hypopituitarism, in particular GHD.
- ***Traumatic brain injury (TBI).*** PRL can be either increased or decreased as a result of TBI [84], however small and diverging data exist at this regard. In a screening study in 509 patients with TBI or subarachnoid hemorrhage (SAH), set to determine the prevalence of post-traumatic hypopituitarism, PRL was measured in 93% and low PRL (defined as PRL < 2.8 ng/ml in females and < 2.1 ng/ml in males) was observed in 1 case, resulting in a 0.2% rate [85]. Among 50 patients with TBI studied by Bondanelli et al., 4 exhibited low PRL levels (defined as PRL < 4 ng/ml for females and < 2 ng/ml for males) resulting in an overall 8% rate of PRL deficiency [86]. These divergent data could be the result of both selection and information bias within studies that do not allow for direct comparison. However, it should be noticed that most disturbances of PRL levels post-TBI will manifest as hyperprolactinemia, due to inhibition of transport of PRL inhibitory factor down the pituitary stalk into the gland [87].
- ***Autoimmune hypophysitis.*** Hypoprolactinemia has been described in patients with autoimmune hypophysitis in coexistence with multiple anterior pituitary hormone deficiencies, yet the condition of low, normal, or elevated PRL levels have been described in this setting [88]. In a review analysis of 379 cases, PRL levels at presentation were low in 25% of patients with lymphocytic adenohypophysitis, none of those with lymphocytic infundibuloneurohypophysitis and 16% of those with lymphocytic panhypophysitis [89]. Remarkably, hypoprolactinemia has also been observed in patients with secondary hypophysitis prompted by immune checkpoint inhibitors used for cancer therapy. These agents are prone to immune-related adverse events (IRAEs). In a combined analysis of 472 patients developing autoimmune hypophysitis during treatment with ipilipumab, an IgG1 monoclonal antibody directed against the inhibitory molecule cytotoxic T-lymphocyte antigen-4 (CTLA-4), low PRL levels occurred in 61.3% of those with hypophysitis [90]. In such disorders, however, hypoprolactinemia does not seem to reflect the extent of pituitary damage.

## Effects of radiotherapy on prolactin secretion and its potential relationship with hypopituitarism

Data on the effect of RT on PRL secretion are limited and heterogeneous, with different patterns of PRL behavior described after RT across the different studies (summarized in Table 2) [48, 56, 58, 91–94]. Radiation may induce the damage of hypothalamic or portal vessels which subsequently leads to hyperprolactinemia due to a reduction in dopamine secretion [25]. This phenomenon is well-recognized following external irradiation and is particularly evident in adult females receiving radiation doses above 40 Gy, with a mild to moderate increase in PRL levels being reported in 20–50% of patients [91]. Noteworthy, unlike post-RT changes affecting the other pituitary trophins, the development of hyperprolactinemia following external beam irradiation seems strongly related to the biological effective dose [91]. Long-term follow-up studies have shown that the PRL peak may occur 1–16 years after RT: once the PRL peak is reached, there is a subsequent progressive decline in PRL even below normal levels, reflecting a time-dependent, slowly evolving direct radiation-induced damage to the pituitary lactotroph [92]. Otherwise, Clark et al. showed a lack of increase in serum PRL and the retained PRL responsiveness to TRH in acromegalic patients treated by interstitial irradiation with yttrium-90, suggesting that PRL secretion is less affected when irradiation is highly localized and not extended to the hypothalamus [93].

**Table 2:** Prolactin disorders after RT and association with other hormone pituitary deficits

Author, year	Reason for RT	Type of RT	Cohort size (number of patients)	Prolactin disorder (%)	Other hormone pituitary deficits*
**Agha A. et al.**,** 2005** [91]	Non-pituitary brain tumors	cEBRT	56	- Hyperprolactinemia in 32% of patients - PRL deficiency in 1 patient	- Panhypopituitarism (1 patient)
**Littley M.D. et al.**,** 1991** [92]	Pituitary tumors	cEBRT	58	- PRL deficiency in 14% of patients treated with RT alone	NA
**Clark A.J. et al.**,** 1983** [93]	Pituitary tumors (Acromegaly)	Yttrium implant	16	- Fall in PRL in 5 hyperprolactinemic patients - No change in normoprolactinemic patients	- Hypopituitarism: 20%
**Toledano Y. et al.**,** 2007** [58]	Pituitary and non-pituitary brain tumors	NS	100 (26 treated with RT)	- PRL deficiency in 19% of patients treated with RT - Severe PRL deficiency in 13% of patients treated with surgery + RT	- GHD: 54–100% - ACTH deficiency: 46–86% - TSH deficiency: 38–100% - Gonadotropin deficiency: 77–79% - DIC: 23–43%
**Mukherjee A. et al.**,** 2003** [56]	Pituitary and non-pituitary brain tumors, ALL	cEBRT, stereotactic RT, Yttrium implant	369 (288 treated with RT)	- PRL deficiency in 3% of patients who received other treatment + RT and in 1.5% of patients treated with RT alone	- GHD: 100% - ACTH deficiency: 46% and 100% in patients with and without CD, respectively - TSH and gonadotropin deficiency: 100% in both patients with and without CD - DIC: 46% and 22% in patients with and without CD, respectively
**Follin C. et al.**,** 2013** [94]	ALL	cEBRT	44	- Severe PRL deficiency in 52% of patients	- GHD: 100%
**Uzun I. et al.**,** 2024** [48]	Pituitary tumors, craniopharyngioma	NS	48 (5 treated with surgery + RT)	- PRL deficiency in 100% of patients treated with surgery + RT	- GH, ACTH, TSH, and gonadotropin deficiency: 100% - DIC: 100% in patients treated with surgery + RT

cEBRT: cranial external-beam radiation therapy; Non-functioning pituitary adenoma: NFPA; CD: Cushing disease; Central diabetes insipidus: DIC; HPA: hypothalamic-pituitary axis; ALL: acute lymphoblastic leukaemia; NS: not specified; NA: not available. * % refer to hormone deficiencies in patients with PRL disorders

PRL deficiency after RT is also reported in literature, ranging from 6 to 19% [56, 58]. Toledano et al. reported that 5 out of 26 patients who underwent RT developed PRL deficiency, which was mild (3–5 ng/ml) in the 2 patients treated with radiotherapy alone and severe (< 3 ng/ml) in the 3 patients treated with both surgery and RT [58]. A high prevalence of PRL deficiency in patients treated with combined surgery and RT was also recently confirmed by Uzun et al. [48]. In this study, median basal PRL levels were significantly lower in patients with panhypopituitarism compared to healthy volunteers (3.36 ng/ml vs. 10.1 ng/ml). Combined surgery and RT were found to be responsible for 10% of cases of hypopituitarism, and all patients receiving combined surgery and RT developed PRL deficiency determined by TRH stimulation test (PRL response < 18.6 ng/ml and < 41.6 ng/ml in males and females, respectively). Furthermore, this study highlighted that PRL deficiency was present in all patients with diabetes insipidus and the etiology of hypopituitarism in these patients was surgery followed by RT in all of them (2 for non-functioning pituitary adenoma, 2 for craniopharyngioma and 1 for Cushing’s disease). The authors therefore suggested that RT was probably associated with a more profound destruction of the anterior pituitary gland, hence PRL deficiency in these patients [48]. Interestingly, Mukherjee et al. found a higher prevalence of PRL deficiency (< 2.35 ng/ml) in patients with Cushing’s disease compared to patients with non-functioning pituitary adenoma [56]. Ten of the 22 patients with reported PRL deficiency were treated with RT alone or in combination with surgery. PRL deficiency occurred after different types of RT, including whole head RT in patients with optic nerve glioma, yttrium implantation in a patient with Cushing’s disease, and stereotactic RT in patients with either craniopharyngioma or Cushing’s disease or non-functioning pituitary adenomas. Moreover, a significant time delay between the administration of RT and the onset of hypoprolactinemia, ranging from 6 to 20 years after treatment, has been reported, suggesting a “late prolactin deficiency” following RT. Of note, this delay in the onset of PRL deficiency has not been reported in patients treated with pituitary surgery alone [56]. This observation is consistent with data from long-term survivors of childhood leukemia treated with cranial RT at doses of 18–30 Gy showing a high prevalence of PRL insufficiency (< 7 ng/ml) up to 20 years after diagnosis (8–27) [94]. In addition, a further decline in basal PRL levels was reported at subsequent 5- (median 11 ng/ml vs. 7 ng/ml) and 8-year follow-up (median 9 ng/ml vs. 5 ng/ml). Interestingly, in this study basal serum PRL levels were positively correlated with age at cranial RT, suggesting that PRL secretion is better preserved when cranial RT is administered at an older age. The authors highlighted that late onset of PRL deficiency had clinical consequences, as seven women became pregnant during the follow-up period and 6 out of 7 women reported failure to lactate. Taken together, these observations suggest a progressive deterioration in lactotrophic function over time following cranial irradiation. Noteworthy, in this population characterized by a high PRL of lactation failure, thyroid function remained normal at baseline and after 16–28 years of follow-up [93, 94].

Although most studies on hypopituitarism have taken in little account PRL deficiency as a clinical entity during patient follow-ups, it can be hypothesized that lower PRL levels indicate a direct radiation damage to lactotroph cells and could reflect an extent damage of pituitary tissue and a higher degree of hypothalamic dysfunction [95]. In fact, Toledano et al. recorded an increasing rate of PRL deficiency with the increasing number of pituitary hormone deficiencies, from none in patients with preserved pituitary function to 38% in patients with four hormone deficits [58]. Similarly, Mukherjee et al. reported that all patients with APD had severe GHD and a variable but consistent association with other pituitary hormone deficiencies [56]. However, the cohorts included in these studies showed heterogeneity in the type, extension and treatment of hypothalamic-pituitary disorders, making it difficult to properly assess the effect of RT on hypoprolactinemia in potential association with hypopituitarism.

It should be also noted that most studies on the effect of RT on pituitary function do not properly assess PRL secretion, as PRL deficiency is usually detected as part of hypopituitarism and not systematically investigated as an isolated disorder, which may lead to an underestimation of hypoprolactinemia after RT. In addition, the often-inadequate follow-up over a long period of time may contribute to the non-recognition of PRL deficiency after RT. Furthermore, the total radiation dose and fraction size, which may influence the development of PRL deficiency, are rarely reported. Finally, a clear definition of PRL deficiency is still lacking, with different criteria used in different studies, making it even more difficult to estimate its true prevalence after RT.

## Clinical relevance

Hypoprolactinemia is an emerging clinical entity that has been increasingly recognized in terms of prevalence and relevance, and needs proper consideration from the endocrine community both when it occurs as an isolated hormone deficiency and when it is part of multiple hypopituitarism. There is evidence that PRL is expressed not only in the pituitary but also in diverse brain areas relating to neurodevelopment, cognition and maternal behaviour (cerebral cortex, hippocampus, amygdala, caudate putamen, brain stem, cerebellum, spinal cord, and choroid plexus), which confirms the pleiotropic actions of this hormone and further stresses the need for attention in patients who undergo cranial RT [36].

PRL is traditionally viewed as the lactotroph hormone, such that agalactia after delivery remains a key symptom to discuss with patients affected by genetic or post-ischemic causes of PRL deficiency [36, 40], as well as with women with a history of cranial irradiation who are searching for a pregnancy. Another condition worth of notice is pregnancy in patients with obesity, since cases of delayed or absent lactation have been reported, possibly due to altered feedback loops related to PRL secretion stimulated by infant suckling. This condition should be adequately addressed in post-partum clinical management, especially when considering the increasing rates of overweight and obesity in pregnancy, particularly in patients subjected to cranial RT [46]. Of some interest, in women with gestational diabetes low PRL levels are reportedly associated with high BMI and, together, these could predict the future onset of T2DM and a worse lipid profile after pregnancy, suggesting that PRL could be an early marker to be inserted in the work-up management of women at risk for metabolic alterations [73]. These results are further corroborated by findings on the dopamine-agonist bromocriptine, which has been found to reduce body weight and improve metabolic disturbances in patients with high PRL levels [96], and has been approved by FDA for the treatment of diabetes [97]. If we consider that metabolic alterations are one of the long-term consequences of RT in cancer survivors [98], the contribution of hypoprolactinemia is still a matter of research.

Similar concerns should be related to the regulation of menstrual cycles and luteal phase since it is necessary to sustain progesterone synthesis by the corpus luteum in the ovary [36, 99], although this aspect could be underestimated in case of concomitant estrogen/progesterone substitutive treatment for multiple hypopituitarism. Furthermore, decreased fertility and early menopause have been observed, indeed all these aspects should be considered in the follow-up of women with low PRL levels [36, 40]. Because PRL is one of the master modulators of IGF-I secretion, as discussed before, when GH secretion is normal but low IGF-I levels are detected as well as when high GH doses are needed to restore IGF-I levels into multiple hypopituitarism, hypoprolactinemia should be taken into account in the clinical picture [2, 40].

By the description of these symptoms, it could appear that hypoprolactinemia is related only to female patients, identifying a gender-related pathology. However, this is not completely correct because PRL receptors are widely distributed and exert many actions, apart from those previously discussed, in both sexes [99]. As described before, abnormalities in the lipid profile, insulin resistance, prediabetes or T2DM, MetS, and MASLD could be diagnosed in any patient with low PRL levels, independently by gender [40]. As a consequence, a strict metabolic follow-up should be performed in patients with hypoprolactinemia. If this is well established in hypopituitarism as suggested by international Guidelines [10], the same management should be considered in isolated low PRL levels. However, what should be investigated is whether a gender-related threshold influences the modulation of PRL on these conditions, since other mediators as transforming growth factor β1 (TGF β1) and activins, apart from estrogens and dopamine, have a role in the sexually dimorphic secretion of PRL [100].

## Conclusions

Hypoprolactinemia represents an underestimated clinical issue that is associated with subtle clinical consequences, which have not been thoroughly characterized. Ideally, we should be able to identify the specific role of hypoprolactinemia in the context of hypopituitarism, and include it in the clinical work-up of patients at risk for hypopituitarism, especially those who underwent RT. Yet, it would be of utmost importance to establish widely accepted appropriate PRL threshold to define hypoprolactinemia, also in relation to gender. RT and more in general any condition determining an extensive the hypothalamus-pituitary represents the main driver of hypoprolactinemia. Therefore, clinicians should be careful in this circumstance to appropriately identify PRL deficiency and follow its potential clinic drawbacks.

## Data Availability

No datasets were generated or analysed during the current study.
